# Interplay Between Vitamin D Levels and Heavy Metals Exposure in Pregnancy and Childbirth: A Systematic Review

**DOI:** 10.3390/pathophysiology31040048

**Published:** 2024-11-21

**Authors:** Tania Flores-Bazán, Jeannett Alejandra Izquierdo-Vega, José Antonio Guerrero-Solano, Araceli Castañeda-Ovando, Diego Estrada-Luna, Angélica Saraí Jiménez-Osorio

**Affiliations:** 1 Área Académica de Enfermería, Instituto de Ciencias de la Salud, Universidad Autónoma del Estado Hidalgo, Circuito Ex Hacienda La Concepción S/N, Carretera Pachuca-Actopan, San Agustín Tlaxiaca P.O. Box 42160, Hidalgo, Mexico; fl467888@uaeh.edu.mx (T.F.-B.); destrada_luna@uaeh.edu.mx (D.E.-L.); 2 Área Académica de Medicina, Instituto de Ciencias de la Salud, Universidad Autónoma del Estado de Hidalgo, Circuito Ex-Hacienda de la Concepción S/N, Carretera Pachuca-Actopan, San Agustín Tlaxiaca P.O. Box 42160, Hidalgo, Mexico; ivega@uaeh.edu.mx; 3 Área Académica de Enfermería, Escuela Superior de Tlahuelilpan, Universidad Autónoma del Estado de Hidalgo, Av. Universidad s/n Centro, Tlahuelilpan P.O. Box 42780, Hidalgo, Mexico; jose_guerrero@uaeh.edu.mx; 4 Área Académica de Química, Instituto de Ciencias Básicas e Ingeniería, Universidad Autónoma del Estado de Hidalgo, Carretera Pachuca-Tulancingo Km 4.5 s/n, Mineral de la Reforma P.O. Box 42184, Hidalgo, Mexico; ovandoa@uaeh.edu.mx

**Keywords:** vitamin D deficiency, pregnancy, heavy metals, toxic metalloids

## Abstract

Background/Objectives: Vitamin D (VD) deficiency has been associated with increased risk of gestational disorders affecting the endocrine system, immune system, and neurodevelopment in offspring. Recent studies have focused on the interaction between toxic elements and micronutrients during pregnancy. This review analyzes the potential relationships between VD levels and heavy metals in pregnant women and their offspring. Methods: A systematic review was conducted according to PRISMA 2020 guidelines, using databases such as PubMed, ScienceDirect, Cochrane Library, and Google Scholar. Boolean operators ‘AND’ and ‘OR’ were applied with terms like ‘pregnancy’, ‘vitamin D’, ‘heavy metals’, and ‘newborns’. Results: From 4688 articles, 14 studies were selected based on relevance and quality. These studies measured the levels of metals like lead (Pb), cadmium (Cd), mercury (Hg), and arsenic (As), in biological samples including maternal blood, umbilical cord blood, placenta tissue, and meconium during different stages of pregnancy, showing an inverse relationship between VD deficiency and heavy metal concentrations, which could be related to the incidence of preterm birth. Conclusions: The review highlights the importance of maintaining adequate VD levels during pregnancy, suggesting that sufficient VD may mitigate the adverse effects of heavy metal exposure, potentially reducing pregnancy-related complications.

## 1. Introduction

Over the past two decades, there has been growing concern about the impact of both VD deficiency and heavy metal exposure during pregnancy on maternal and fetal health. VD deficiency is typically defined by circulating 25-hydroxyvitamin D (25OHD) levels of 30 nmol/L [1]. In recent years, meta-analyses of maternal VD concentrations have consistently confirmed an inverse relationship between maternal 25OHD levels and the risk of gestational complications, including gestational diabetes [2,3,4], preeclampsia [3,5,6,7], anemia [8], preterm birth [9,10], spontaneous pregnancy loss and miscarriage in the first trimester [11,12,13], COVID-19 severity [14] and delivering small-for-gestational-age infants [3,15]. These findings underscore the crucial role of VD in maternal and fetal health, extending beyond its well-established functions in calcium homeostasis and skeletal mineralization.

Multiple factors contribute to VD deficiency, including genetic variants, age, obesity, VD supplementation practices, and sunlight exposure, which is further influenced by factors like season, latitude, skin-covering clothing, skin pigmentation, sunscreen use, and outdoor activity levels [16,17,18,19]. Additionally, environmental pollutants, such as particulate matter (PM2.5 and PM10), have been linked to altered maternal VD status [20,21] and reduced VD levels in newborns [22]. Exposure to endocrine-disrupting chemicals like phthalates and bisphenol A has also been associated with an increased risk of VD deficiency during pregnancy [23] and interference with VD endocrine signaling (VDES) [24].

The rising pollution in industrialized countries has led to increased exposure to toxic metals and metalloids through contaminated water sources used for agriculture and human consumption. This exposure often involves a combination of multiple metals and metalloids, rather than a single element, which can lead to more severe effects due to their synergistic interactions [25]. Previous studies have established a link between such exposure and an elevated risk of spontaneous preterm birth [26]. Caserta et al. [27] emphasized the significant impact of early exposure to heavy metals on placental permeability and neonatal outcomes. Specifically, Pb, Cd, and Hg have been shown to cross the placental barrier, accumulate in amniotic fluid and fetal tissues, and are associated with various adverse infant health outcomes, including endocrine, immune, neurological, and developmental disorders. Additionally, Bauer et al. [28] report that perinatal exposure to metals such as arsenic (As), copper (Cu), manganese (Mn), and selenium (Se) may contribute to behavioral development abnormalities in offspring.

Recent studies have consistently reported a strong association between prenatal Pb exposure and VD deficiency [29]. However, the existing literature lacks a comprehensive understanding of how VD status interacts with this topic and remains limited, underscoring the need for further investigation into whether there is a positive or negative relationship between VD levels and the presence of heavy metals and metalloids in pregnant women. In this context, the aim of this systematic review is to examine the available clinical evidence on the interaction between VD and heavy metals/metalloids, with a particular focus on their potential impact on maternal and offspring health.

## 2. Materials and Methods

### 2.1. Study Protocol

This systematic review was conducted in accordance with the Preferred Reporting Items for Systematic Review and Meta-Analyses (PRISMA) guidelines [30]. The protocol for the review was registered in the Prospective International Registry of Systematic Reviews (PROSPERO) under registration number CRD42024575680 on 27 August 2024. Three authors participated in the database search and in establishing the inclusion and exclusion criteria. All authors equally contributed to assessing the selected articles and extracting data.

### 2.2. Eligibility Criteria

The eligibility criteria were established by the corresponding authors (T.F-B and A.S.J-O.). During the selection process, four team members (T.F-B., J.A.G-S., J.A.I-V., and A.S.J-O.) independently searched databases using keywords and reviewed the titles and abstracts of retrieved references to identify relevant studies. Inclusion criteria focused on original human studies evaluating the impact of heavy metals and VD levels in pregnant women and their offspring. Eligible studies included the analysis of biological samples, pregnancy pathology, vitamin D supplementation, and the presence of at least one heavy metal or metalloid, as well as studies involving neonates and offspring, with no restriction on publication years. Animal studies, chronic degenerative diseases, studies on pregnant minors, or studies unrelated to pregnancy were excluded. The selected studies were validated by all authors, and in case of disagreement, a working meeting was convened to reach a consensus.

### 2.3. Search Strategy

Our systematic review included case–control, cohort, observational, and controlled clinical trials that examined the effects of vitamin D and heavy metal/metalloid levels in pregnant women and their offspring. A systematic search was conducted through 3 August 2024, across multiple databases, including PubMed, ScienceDirect, Cochrane Library, and Google Scholar. The search strategy combined free-text terms and keywords “Vitamin D status” “Vitamin D” “25-hydroxyvitamin”, “Pregnancy”, “Heavy Metals”, “Metal concentrations” and “Metalloids”, utilizing Boolean operators (AND/OR) for optimal term combinations. For instance, search queries included (((“Vitamin D” [Mesh]) AND “pregnancy” [Mesh]) AND “Metals, Heavy” [Mesh]) OR “Metalloids” [Mesh]). During the search process, no date restriction and filters by item type were applied.

### 2.4. Study Quality Assessment

Study quality was assessed by two reviewers (T.F-B and A.S.J-O.) using the Joanna Briggs Institute (JBI) checklist [31], which is specifically designed for case–control, cohort, cross-sectional analytical, and randomized controlled trials. This checklist evaluates the risk of bias through 10 questions covering criteria such as sample selection, exposure assessment, risk of confounding, and the appropriateness of the statistical analysis. Each question was answered “yes”, “no”, “unclear”, or “not applicable”, and the percentage of “yes” responses was used to determine the overall quality of each study.

The quality of the articles was categorized into three levels:

- Q1 (high quality, low risk of bias): ≥75% “yes” responses;
- Q2 (moderate quality, unclear risk of bias): ≥50–74% “yes” responses;
- Q3 (low quality, high risk of bias): ≥50–74% “no” responses.

Out of the 17 included studies, 10 were classified as high-quality (Q1), 4 as moderate-quality (Q2), and 3 as low-quality (Q3). Studies classified as low-quality were considered to have a high risk of bias and were therefore excluded from the final analysis. Only studies with adequate quality and a lower risk of bias were included in the review.

### 2.5. PECO Statement

A PECO (Population, Exposure, Comparator, and Outcome) framework was employed to formulate the research question, as detailed in Table 1, This approach provided a clear definition of the systematic review’s objectives, the search terms utilized, and the inclusion and exclusion criteria for studies related to the association of vitamin D and heavy metal levels during pregnancy and childbirth in the mother and offspring.

### 2.6. Data Extraction

Three reviewers (T.F-B., J.A.G-S., and A.S.J-O.) reached a consensus on the extracted data, which included title and year, study design, country, number of samples, biological sample and analysis, stage of pregnancy, VD supplementation or VD concentration, heavy metal/lloid evaluated, and significant findings. The research team conducted the comprehensive data analysis to perform a quantitative analysis of the effects. However, due to substantial heterogeneity among the elements evaluated during pregnancy, we opted to present this analysis as a systematic review.

## 3. Results

### 3.1. Study Selection and Characteristics

**Figure 1:**
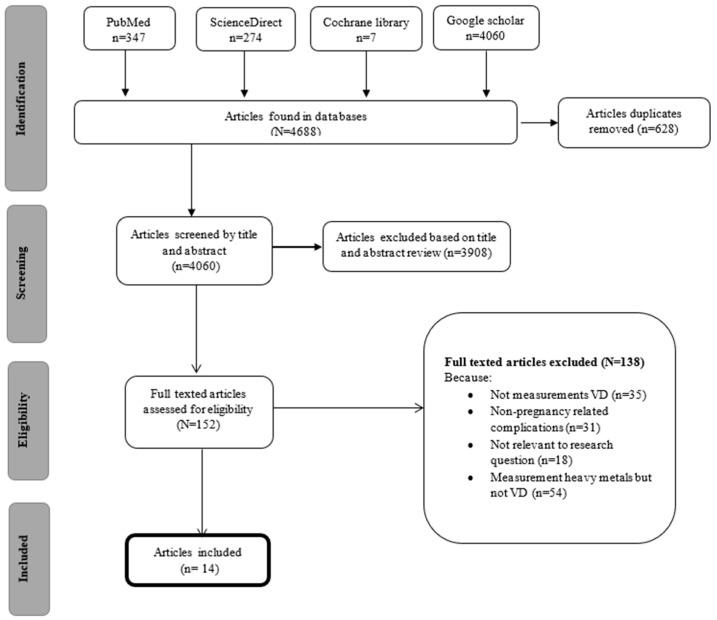
PRISMA flow diagram of the systematic review.

A comprehensive search of multiple databases, including PubMed, ScienceDirect, the Cochrane Library, and Google Scholar, was conducted, yielding a total of 4688 initial records: 347 from PubMed, 274 from ScienceDirect, 7 from Cochrane Library, and 4060 from Google Scholar. The search spanned from 3 August to 2 September 2024. After removing 628 duplicate records, 4060 unique studies were selected based on their titles and abstracts. From these, 152 studies were further evaluated in full-text form (Figure 1).

Applying predefined inclusion and exclusion criteria, 138 studies were excluded for the following reasons: 35 did not assess VD levels, 31 focused on non-pregnancy-related complications, 18 were considered irrelevant to the research question, and 54 measured heavy metals without corresponding VD measurements. Consequently, 14 full-text articles were assessed for eligibility.

Ultimately, the 14 studies met all inclusion criteria and were included in the final review. The study selection process, including the number of studies at each stage and the reasons for exclusion, is detailed in the PRISMA flow chart presented in Figure 1.

This review incorporated fourteen studies, encompassing diverse methodological designs: seven cohort, three cross-sectional, three observational, and one randomized clinical trial. The studies were geographically distributed across eight countries, with a preponderance in China, Canada, and Turkey. Participants included pregnant women, newborns, and, in some cases, mother–infant dyads. Exposure to heavy metals varied, encompassing both contaminated drinking water and unspecified environmental contaminants. Biological samples analyzed included blood, urine, meconium, and placenta. The primary analytical methods employed were Liquid Chromatography–Tandem Mass Spectrometry (LC-MS/MS), Inductively Coupled Plasma Mass Spectrometry (ICP-MS), and High-Performance Liquid Chromatography (HPLC). A total of 12,508 individuals contributed biological samples across the 14 included studies, summarized in Table 2.

### 3.2. Associations Between VD Levels and Heavy Metal Exposure During Pregnancy

The data on VD and metal measurements across studies are highly heterogeneous. Some studies measure serum VD concentrations using analytical methods such as immunoassays (used in six studies), mass spectrometry (used in four studies), and high-performance liquid chromatography (HPLC, used in three studies). Others estimate VD intake through food frequency questionnaires (used in five studies) or environmental exposure questionnaires (used in three studies). Additionally, maternal blood was analyzed in ten studies, umbilical cord blood (UCB) in five studies, and placentas in three studies. Only one study evaluated the effect of VD supplementation [38]. A recent study from 2024 was the only one that analyzed the relationship between VD levels and five As species in pregnant women during the late second and early third trimesters, using urine and blood samples. The results indicated that VD deficiency was associated with higher As concentrations. Although Pb and Cd are the most commonly evaluated metals in a single sample, recent studies have begun measuring multiple metals in a single sample, including venous blood, urine, UCB, placenta, and meconium across different gestational trimesters [32].

In the first trimester of gestation, Ti, Co, Cu, As, Se, Hg, and Tl were detected in 95% of maternal urine samples, while Ti, Cu, As, and Se were found in 98% of samples in a cohort from Nanjing Maternity and Child Health Care Hospital (Nanjing, China). Interestingly, it was determined that the relationship between Co levels and dental eruption timing is modified when the mother is not supplemented with VD during pregnancy (beta-value: 0.57, *p* = 0.016) [37].

In the middle of the gestational period, between the first and second trimesters, it has been observed that blood levels of Zn, Co, and VD decreased in pathologies such as hydatidiform mole (CHM), especially when Cd concentrations increased significantly [44]. In the LIFECODES cohort, higher VD levels in the first trimester were positively associated with lower urinary Pb and Se levels and were influenced by factors such as race. The mean VD level was 26 ng/mL, with 34% of women exhibiting low levels. Additionally, mean heavy metal concentrations were higher in women with VD levels below 20 ng/mL [35].

A study exploring the genetic basis of Pb toxicity in pregnant women identified an association between specific VD receptor (VDR) variants and lower Pb levels, suggesting that VD metabolism may play a protective role against Pb toxicity during pregnancy. The H8 haplotype, which combines alleles f, a, and b, was particularly associated with reduced Pb exposure in pregnant women. This finding could help identify individuals who are genetically less susceptible to Pb-related health risks [43]. Therefore, it is advisable to consider the presence of concomitant diseases in the design of future studies, as they may modify the direction of the results.

The most robust data comes from studies that evaluate both metal and VD levels throughout pregnancy. The MIREC study (Maternal-infant research on environmental chemicals), which involves ten cities across Canada, evaluated Pb, Cd, and Hg in maternal blood, UCB, and meconium. The findings revealed that these metals were detectable in maternal blood at both the beginning and end of gestation. VD concentrations were generally moderate to high, while heavy metal levels were elevated in the first trimester compared to the third. Exposure to Pb, As, and even minor increases in these metals were linked to an increased risk of preterm birth (PTB), and spontaneous preterm birth (SPTB). Participants with VD levels below 40 nmol/L exhibited a higher risk of PTB and SPTB, whereas levels above 50 nmol/L mitigate this risk, suggesting a protective role for VD against the adverse effects of blood Pb [33].

In another sub-analysis of the MIREC cohort, the directionality of the association between VD and toxic metals was evaluated using multivariate linear regression to determine if Cd and Pb are cross-sectionally and longitudinally associated with VD concentrations during pregnancy, adjusting for confounding variables such as smoking, a source of Cd. The analysis also included sociodemographic variables, parity, pre-pregnancy, and study site, as well as time-varying covariates across pregnancy, such as season, source of VD, and time spent outdoors. Cd concentrations in the first and third trimesters of pregnancy were inversely associated with VD, with the strongest association found at delivery, and this relationship was reduced by smoking status. Bidirectional analysis showed that VD in the first trimester predicts Cd changes in the third trimester. This suggests that blood metal levels at the end of pregnancy could be modified by initial VD concentrations [34]. In another MIREC cohort, Cd, Pb, Mn, and Hg concentrations were analyzed in the first trimester of pregnancy. Detectable levels of heavy metals were found in most maternal blood samples. Strong correlations were observed between metal concentrations in maternal blood and UCB except for Cd. Higher VD levels were linked to lower levels of Cd, Pb and Mn in maternal blood and Pb in UCB blood [42].

The findings from the South American cohort indicate that lithium (Li) exposure in water may negatively affect VD absorption during pregnancy. The study observed a correlation between blood and urinary Li levels, both of which increased with gestational age. Notably, the average VD concentration was found to be 41 nmol/mL, with a significant percentage of women (58%) having VD levels below 50 nmol/L and 19% below 30 nmol/L. Furthermore, the study concluded that seasonal variation did not significantly alter the relationship between VD and Li exposure (summer/fall versus spring/winter). An increase of 25 μg/L in blood Li levels was associated with 3.5 times higher likelihood of having VD concentrations below 50 nmol/L and 4.6 times higher likelihood of having VD levels below 30 nmol/L [41].

In another study analyzing serum, placental, and UCB samples from women who gave birth, it was found that placental samples from term newborns had higher concentration of VD and Mn, along with lower levels of Hg and Pb compared to preterm newborns. Despite these differences, both groups exhibited VD levels below the normal range, with placental VD concentrations notably higher in term newborns than in preterm ones. Additionally VDD was linked to adverse pregnancy outcomes, such as intrauterine growth restriction and preterm birth [40].

Moreover, a study in Bangladesh [38] investigated the effects of VD supplementation during pregnancy on metal distribution. The study administered VD_3_ doses of 4200, 16,800, or 28,000 IU starting in the second trimester. The results indicated that maternal and neonatal UCB concentrations of Pb and Cd were 6 to 7.4% higher in the VD supplementation groups compared to the placebo group, although the confidence intervals included the null value. Higher levels of VD supplementation (16,800 and 28,000 IU) were associated with an increased probability of detecting Pb and Cd in the UCB. The study emphasized the need for further data on calcium supplementation and the source of metals or time of exposure.

## 4. Discussion

This review aimed to examine the available clinical evidence on the interaction between vitamin D and heavy metal/lloids, focusing on their potential impact on maternal and newborn health. By excluding animal models, we sought to reduce the risk of dose transfer bias between laboratory animals and humans.

During pregnancy, the fetus depends on the mother for VD supply through the UCB [46], and prenatal VD supplementation is associated with neonatal VD levels [47]. Therefore, maintaining adequate VD during pregnancy is not only essential for maternal health but also for fetal development. Natural sources of VD include ergocalciferol from plants and VD_3_ from sunlight exposure and animal products like eggs, fish, and meat. However, insufficient nutritional intakes among pregnant women are a recognized issue in many countries [48], making supplementation important to meet the recommended intake of VD and other micronutrients [49]. The recommended VD supplementation during pregnancy ranges from 600 to 2000 IU per day, depending on baseline levels and risk factors for deficiency [50].

Various external factors, such as latitude [51,52,53], race, ethnicity, skin color [54,55,56,57,58], and cultural practices that limit sun exposure, also contribute to VD deficiency and insufficiency, which remain significant public health concerns. Clinical studies often define VDD, insufficiency, and sufficiency by measuring circulating 25OHD levels [59,60]. According to the Institute of Medicine (IOM), 25OHD levels between 20 and 30 nmol/L are considered deficient, 31 to 50 nmol/L as insufficient, and levels above 50 nmol/L as sufficient. However, these definitions are not universally accepted. The Endocrine Society Practice Guideline [61], for instance, defines deficiency as concentrations below 50 nmol/L, insufficiency as 52.5 to 72.5 nmol/L, and sufficiency as 75 to 250 nmol/L. This discrepancy highlights the ongoing debate surrounding optimal VD levels, particularly during pregnancy. While there are no specific guidelines for VD levels in pregnancy, it is generally accepted that maintaining a 25OHD concentration of at least 50 nmol/L would meet the needs of 97.5% of the population. However, recent studies have emphasized the influence of environmental interactions between prenatal exposure to heavy metals and reduced maternal VD levels, both of which may exacerbate pregnancy complications and affect child health outcomes. In recent years, the impact of environmental conditions on VDD risk has gained recognition [52,62,63,64].

On the other hand, Pb is a well-known neurotoxicant. During pregnancy, the fetus requires calcium, and hormonal changes can cause Pb to be released from the mother’s bones [65]. Even in the absence of direct maternal exposure, women living in areas with higher Pb levels face a greater risk, and Pb can cross the placental and blood–brain barrier, affecting fetal blood and potentially altering DNA methylation, histone protein modifications, and microRNA expression. Pb exposure during pregnancy is associated with low birth weight, premature birth, behavioral and learning problems, and an increased risk of ADHD, autism, heart disease, cancer, and Alzheimer’s disease in offspring [66,67,68]. Certain dietary patterns, such as those high in leafy greens, have been linked to lower blood Pb concentrations in pregnant women [69].

Cd is recognized as a toxic metal that contributes to carcinogenesis and damages multiple organs and tissues. The United States Environmental Protection Agency classifies Cd as a probable human carcinogen (Group B1). Research indicates that Cd can be absorbed through various metal transporters including the calcium channel (TRPV6), zinc transporter 2 (ZnT2), iron-regulated transporter type proteins ZIP (ZIP-14), and proton-coupled divalent metal transporter (DMT-1) [70,71]. Exposure to Cd during pregnancy is linked to altered gene expression in the embryo, which can restrict fetal growth and development, leading to long-term organ dysfunction. Additionally, Cd affects placenta formation and function and is associated with epigenetic modifications, such as abnormal methylation [72,73,74]. It has been detected in UCB at lower concentrations than in maternal blood, and its presence is associated with low birth weight in offspring [75].

Hg in both elemental and organic forms can cross the placenta during pregnancy, posing risk to the developing fetus [76]. Methyl-Hg (MeHg) binds to sulfhydryl groups of cysteine, and due to its structural similarity to methionine, enters cells through amino acid transporters and binds to glutathione [77,78]. Placental transporters, including amino acid transporters LAT1 and rBAT, are involved in the uptake of MeHg in the placenta [79]. Hg concentrations in the placenta correlate with those in fetal organs and are linked to cognitive and language impairments, neural tube defects, and an increased risk of autism and ADHD [80,81].

As, a naturally occurring metalloid, is one of the primary contaminants found in drinking water worldwide, in its inorganic form arsenate (pentavalent As) and arsenite (As^3+^). As is a known carcinogen commonly present not only in drinking water, but also in various foods such as rice, vegetables, fruits, meats, and dairy products [82,83]. Once ingested, As is metabolized via glutathione S-transferase omega-1 (GSTO1), which utilizes glutathione (GSH) as a reducing agent, and As methyltransferase (AS3MT), which facilitates both oxidation and methylation using S-adenosylmethionine (SAM) as a methyl donor. This process leads to the sequential methylation of arsenic into mono-, di-, and trimethylated species, with continuous interconversion between oxidized and reduced forms following ingestion [84].

The U.S. Environmental Protection Agency (EPA) has set a maximum permissible limit of As in drinking water at parts per billion levels [85]. The potential impact of As exposure on pregnancy outcomes remains a topic of debate [86,87]. Some studies suggest a link between high As exposure during pregnancy and adverse effects such as PTB, low birth weight, gestational diabetes, infant mortality, and preeclampsia [88,89,90]. However, much of this research has been conducted in populations with high exposure levels, and there are concerns that As concentrations measured in urine during the first trimester may not accurately reflect exposure during critical physiological periods. Higher exposure levels may occur in the second and third trimesters, yet changes in glomerular filtration rate, plasma volume expansion, and increased methylation efficiency during pregnancy could introduce biases in exposure assessment. Moreover, there are limited data available on As exposure across the entire gestational period [91].

The prevalence of VDD in various populations and its association with diseases underscore the importance of understanding the underlying mechanisms. In particular, regarding maternal exposure to metals and its impact on UCB, VD remains a significant, yet poorly understood, area of research. Since the 1990s, it has been suggested that VD could increase the absorption of toxic metals, such as Pb and Cd, through calcium metabolism. Studies in chicks treated with cholecalciferol showed that calcium and phosphorus levels led to increased absorption of Pb and calcium, with VD-induced calcium-binding protein (CaBP) playing a role [92]. In children, blood Pb levels have been observed to rise during the summer, coinciding with increased VD synthesis [93].

Heavy metals have been shown to downregulate the transcription of cytochrome P450 mixed-function oxidases (CYPs), which are essential for various metabolic processes, including VD metabolism. Recent studies in mice that were fed organic calcium indicated lower mRNA levels of calcium transporters in the duodenum, correlating with reduced concentrations of Pb, Cd, As, parathyroid hormone expression, renal CYP27B1 activity, and 1,25(OH)2D3 levels. The CYP27B1 enzyme, expressed in the kidneys, placenta, and macrophages, plays a crucial role in the metabolism of VD [94]. In experiments involving rats exposed to Cesium 137, an increase in 1,25(OH)2D3 levels was observed without corresponding changes in plasma VD levels, and this was accompanied by decreased expression of cyp2r1 and cyp27b1 mRNA, despite the VD receptor mRNA levels remaining unchanged [95,96].

Polymorphisms in the CYP2R1 gene have been linked to variations in VD levels and conditions such as spontaneous abortion and gestational diabetes mellitus [97,98]. The CYP2R1 enzyme, which is expressed in the liver, is responsible for VD 25-hydroxylase activity [99]. Disruptions in the production of cholecalciferol can impair the absorption of VD, and genetic modifications in liver and kidney 25-hydroxylase can lead to decreased serum VD levels. Therefore, comprehensive analyses of the genome, transcriptome, and proteome are essential for understanding the genetic factors that influence the response to VD.

Another critical aspect to consider is the potential inverse relationship between heavy metal exposure and placental homeostasis. This disruption is thought to arise from oxidative stress, altered glutathione metabolism, and inhibition of Na+/K+-ATPase activity [100]. Since VD biosynthesis relies on hepatic metabolism, oxidative damage to mitochondria and the endoplasmic reticulum may explain the associations observed in longitudinal studies, leading to the suppression of VD_3_/D_2_ hydroxylation to 25OHD in the liver [59,101]. Given these complex interactions, animal studies are vital for investigating the effects of VD on the metabolism of metals and metalloids.

### 4.1. Limitations

The methodological differences between the studies included in this review present significant limitations for interpreting the results regarding the relationship between VD levels and heavy metal exposure during pregnancy. Variations in the biological material analyzed, such as maternal blood, UCB, placental tissue, and meconium, can influence the accuracy and comparability of the findings. Additionally, the considerable variability in sample size across studies may affect the generalization of the results. The geographical diversity of the studies, which spans different countries and environmental contexts, introduces further variability in heavy metal exposure and VD levels, complicating the interpretation of the observed associations. These methodological differences highlight the need for more homogeneous and standardized research to draw more robust conclusions about the relationship between VD levels and heavy metal exposure during pregnancy.

### 4.2. Future Directions

Based on current clinical evidence, animal studies are necessary to determine the causal relationships between VD and exposure to toxic materials. The limited number of available animal studies means that these relationships cannot be fully understood through clinical studies alone. Furthermore, the absence of preclinical research specifically addressing the impact of VD_3_ supplementation on the metabolism of toxic metals highlights a significant gap in our knowledge. This area of investigation holds the potential to answer fundamental questions posed by clinical researchers and physicians, particularly regarding whether VD influences the absorption of heavy metals during pregnancy.

The availability of various maternal and child cohorts worldwide, which provide continuous monitoring of pregnancy progression, suggests a promising future for clinical research in this area. Before implementing VD supplementation guidelines, it is crucial to thoroughly investigate the dynamics of heavy metals in maternal blood and UCB. Although some studies have explored the directionality of the relationship between VD and metal concentrations, there is a notable lack of studies that examine metal levels at different stages of pregnancy. However, the advancement of omics sciences, particularly metalloids, offers new possibilities. This technology enables the simultaneous evaluation of various metals and metalloids in a single run, which is especially advantageous when analyzing biological samples from newborns. For example, metabolomics is currently a powerful tool for monitoring metalloids in biological samples. It allows the identification and quantification of metabolites that can serve as biomarkers of heavy metal exposure, offering a comprehensive approach to assess metalloid toxicity and facilitating the early detection of adverse health effects [102]. Recent studies have shown that the application of metabolomics can help identify metabolite patterns associated with heavy metal exposure, which could be crucial for developing intervention and prevention strategies in public health [103].

Additionally, collecting comprehensive data on factors that modulate the VD response, including prior exposure to heavy metals, through detailed questionnaires is vital for multivariate analyses. This approach could help clarify the relationship between VD and heavy metal concentrations. Current evidence suggests a significant role of maternal VD levels early in pregnancy in reducing heavy metal concentrations at term, a finding that warrants further investigation in future studies.

Finally, we consider it relevant to propose some concrete measures to reduce the impact of heavy metals on the health of pregnant women and their children. Firstly, it is important to promote a nutrient-rich diet that includes foods that may help reduce the absorption of heavy metals, as well as supplements with VD. It is also suggested to educate and raise awareness among pregnant women through the implementation of educational programs and various types of informational materials to inform them about the risks associated with heavy metal exposure and common sources of contamination. Additionally, we believe it is essential for governments to ensure access to safe drinking water through monitoring and purification systems, as well as the development of policies that regulate the use of heavy metals in consumer products to minimize exposure. Finally, we hope that healthcare systems will incorporate screening programs to identify pregnant women with elevated levels of heavy metals within their routine medical control protocol for pregnant women, to promote the implementation of measures or treatments if necessary.

## 5. Conclusions

The presented review studies reveal diverse patterns in the relationship between VD levels and exposure to heavy metal/lloids during pregnancy, with variations depending on the trimester of gestation and the type of metal analyzed. Current evidence is highly heterogeneous, with differences in methods used to measure VD and metals. While some studies suggest that VDD is associated with higher concentrations of Pb, As, and other metals, the data are inconsistent and limited. Moreover, while VD supplementation during pregnancy shows effects on metal distribution, it has not been thoroughly investigated. The robust studies, such as the MIREC cohort, indicate that higher VD levels are associated with lower concentrations of certain metals like Pb, Cd and Mn in maternal blood and UCB, suggesting a potential protective role of VD. However, the variability in results underscores the need for further studies that consider factors such as genetics, prior metal exposure, and VD supplementation.

The relationship between VD and metal toxicity during pregnancy remains a critical area of research with significant implications for maternal and fetal health. Continued investigation is essential to clarify these mechanisms and develop appropriate interventions to protect the health of mothers and their offspring.

## Figures and Tables

**Table 1:** PECO statement in this systematic review.

PECO	Evidence
Population	Pregnant women and infants
Exposure	Exposure to naturally occurring heavy metal/lloids, or toxic environmental contaminants.
Comparator	Comparison of metal and metalloid concentrations in biological samples with vitamin D levels in pregnant women and infants.
Outcome	Relationship or association between vitamin D levels and heavy metal concentrations in mothers and infants, as well as complications.

**Table 2:** Clinical studies examining the relationships between VD and heavy metal exposure during pregnancy in mother–infant pairs.

Ref.	Title and Year	Study Design	Country	Sample	Biological Sample/Analysis	Stage of Pregnancy/Delivery	VD Concentration/Supplementation	Heavy Metal/Lloid Evaluated	Significant Findings	Risk of Bias (Low, Medium, High)
[32]	Association between urinary arsenic species and vitamin D deficiency: a cross-sectional study in Chinese pregnant women (2024)	Cross-sectional	China	Pregnancy (n = 391)	Urine/heavy metals (HPLC and ICP-MS) Blood/VD (LC-MS/MS)	Second and third trimester	No supplementation Concentrations: <12 ng/mL (n = 60)	Species to As (As^3+^, As^5+^, MMA, DMA and AsB)	Subjects with higher levels of As^3+^ were more likely to develop VDD. Also, those with VDD showed significantly higher concentrations of As^3+^ and DMA compared to subjects without deficiency	Low
[33]	Association between toxic metals, vitamin D and preterm birth in the maternal-infant research on environmental chemicals study (2023)	Cohort	Canada	Pregnancy (n = 1851)	Blood/heavy metals and VD (SG)	First and third trimester	No supplementation Concentration: The mean 25OHD level was first trimester (69.7 nmol/L) and third trimester (78.4 nmol/L)	Cd, As, Pb and Hg	In pregnant women, blood Pb levels were generally low; a positive correlation was found between elevated blood Pb and an increased risk of PTB. VD levels may influence this relationship, as the risk of SPTB was significantly higher when elevated concentrations of Pb and As were observed, along with a decrease in VD levels below 50 nmol/L.	Low
[34]	Blood metals and vitamin D status in a pregnancy cohort: a bidirectional biomarker analysis (2022)	Cross-sectional	Canada	Pregnancy (N = 3554)	Blood/heavy metals and VD (ELISA and LIAISON, LC-MS/MS)	First trimester (n = 1905) Third trimester (n = 1649) Delivery (n = 1542)	No supplementation Concentration: ≥50 nmol/L First trimester (85% participants) Third trimester (87% participants) Delivery (84% participants) <30 nmol/L (4% participants)	Pb and Cd	VD concentrations were associated with lower metal concentration in blood during the third trimester, with a 9% reduction in Cd and a 3% reduction in Pb. These findings suggest that VD may play modulatory role in regulating Cd and Pb concentrations during pregnancy	Low
[35]	A prospective study of maternal 25-hydroxyvitamin D in the first trimester of pregnancy and second trimester heavy metal levels (2021)	Cohort	Boston	Pregnancy (n = 322)	Blood/VD (LIAISON) Urine/heavy metals (SG)	First and second trimester	No supplementation The mean 25OHD was 26 ng/mL (34% presented low levels in first trimester)	As, Ba, Be, Cd, Hg, Pb, Sn, Tl, U, W, Cu, Cr, Mn, Mo, Ni, Se and Zn (second trimester)	Pregnant women with low VD levels exhibited significantly higher concentrations of Pb, Sn and W. In particular, Pb concentrations were 54% higher, and the detection rate of W was 1.58 times greater in women with insufficient VD levels compared to those with adequate VD levels.	Low
[36]	Association of urine metals and mixtures during pregnancy with cord serum vitamin D levels: A cohort study with repeated measurements of maternal urinary metal concentrations (2021)	Cohort	China	Mother–newborn pairs (n = 598)	Mothers: urine/VD (ICP-MS) UCB/heavy metals (LC-MS/MS)	All trimesters of pregnancy and delivery	No supplementation The mean 25OHD was 16.76 ng/mL	Al, V, Cr, Mn, Co, Ni, Cu, Zn, As, Se, Rb, Sr, Ag, Cd, Cs, Ba, Tl, Pb, Th and U	Maternal exposure to Co, Tl and V during pregnancy is associated with decreased total VD levels in UCB, heightening the risk of neonatal VDD. Moreover, combined exposure to these metals shows a synergistic effect on VD status, suggesting a higher risk of deficiency in newborns exposed to multiple contaminants.	Low
[37]	A metabolomic study on the association of exposure to heavy metals in the first trimester with primary tooth eruption (2020)	Cohort	China	Pregnancy (n = 244) Infants (n = 183)	Urine/heavy metals (ICP-MS) Oral examination	First trimester	Supplementation: Pregnancy (n = 88) Infants (n = 171) Concentration: not reported	Ti, V, Fe, Co, Cu, As, Se, Cd, Sn, Hg, Tl and U (Pregnancy)	No significant associations were identified between heavy metal exposure during the first trimester and the eruption of primary teeth, except for Co, Cd and As. Higher Co concentrations were positively linked to delayed eruption of the first tooth and negatively associated with the number of teeth at one year of age, as well as with VD supplementation.	Medium
[38]	Vitamin D treatment during pregnancy and maternal and neonatal cord blood metal concentrations at delivery: results of a randomized controlled trial in Bangladesh (2020)	Randomized controlled trial	Bangladesh	Pregnancy (n = 619) Newborns (n = 516)	Maternal and UCB/VD and heavy metals (ICP-MS and LC-MS/MS)	Delivery	Supplementation: groups (4200, 16,800 and 28,000 UI VD_3_ for week/second and third trimesters) The median 25OHD level was 25 nmol/L	Cd, Pb, Hg and Mn	Mean Pb y Cd concentrations in UCB were higher in the supplemented groups compared to the placebo group, particularly those receiving higher VD treatments (16,800 and 28,000 IU).	Low
[39]	The concentration of micronutrients and heavy metals in maternal serum, placenta, and cord blood: A cross-sectional study in preterm birth (2019)	Cross-sectional	Indonesia	Mother–infant pairs (n = 51)	Maternal serum, placenta and UCB/VD and heavy metals (ICP-MS and LC-MS/MS)	Delivery	No supplementation Concentration: not specified	Pb and Hg	Lower concentrations of Cu samples were observed in term infants than in PTB, also associated with lower levels of VD and higher concentrations of heavy metals.	Low
[40]	Selected maternal, fetal and placental trace element and heavy metal and maternal vitamin levels in preterm deliveries with or without preterm premature rupture of membranes (2018)	Cohort	Argentina	PTB with PPROM: (n = 33) PTB without PPROM: (n = 35)	Maternal serum/VD and heavy metals (FAES, HPLC) Placental and UCB/heavy metals (FAES)	Third trimester Delivery	No supplementation Concentration: with PPROM: 0.0116 ± 0.0058 Without PPROM: 0.0163 ± 0.0088	Mg, Pb, Zn, Cd	Compared to PTB with PPROM, it is associated with lower maternal serum Mg concentrations, increased placental Mg concentrations, and reduced Zn concentrations in both maternal and UCB. Additionally, patients with PPROM show elevated levels of VD. Cd and Pb concentrations did not differ between the two groups for maternal or UCB.	Low
[41]	Exposure to lithium through drinking water and calcium homeostasis during pregnancy: A longitudinal study (2016)	Cohort	Argentina	Pregnancy (n = 178)	Urine/heavy metals (ICP-MS and LC-HG) Blood/VD and heavy metals (ICP-MS)	All trimesters of pregnancy	No supplementation The median 25OHD level was 41 nmol/L (58% or the women) 19% had <30 nmol/L	Li, As and Cs	Increased Li concentrations were associated with a fivefold higher risk of having VD concentrations < 30 nmol/L. Low VD levels are detrimental to both maternal and fetal health.	Low
[42]	Maternal and fetal exposure to cadmium, lead, manganese and mercury: The MIREC study (2016)	Cohort	Canada	Pregnancy (n = 1938)	Blood/VD and heavy metals (ICP-MS) UCB/heavy metals (ICP-MS) Meconium/heavy metals (ICP-MS)	First and third trimester Delivery	Supplementation: yes, but not clear Concentrations: not clear	Pb, Cd, Mn and Hg	VD intake was significantly associated with lower levels of Cd, Pb and Mn in maternal blood, as well as reduced Pb levels in UCB.	Low
[43]	Vitamin D receptor haplotypes affect lead levels during pregnancy (2010)	Observational	Brazil	Pregnancy (n = 256)	Blood/VD and heavy metals (PCR) UCB/VD and heavy metals (PCR)	Third trimester Delivery	Supplementation: not reported Concentrations: not reported	Pb	VDR H8 haplotype was associated with lower Pb levels in maternal serum. The H8 and H4 haplotypes were associated with lower %Pb-S/Pb-B ratios than the H1 haplotype.	Low
[44]	Catalase activity, serum trace and heavy metal concentrations, vitamin A, vitamin D and vitamin E levels in hydatidiform mole (2009)	Observational	Turkey	HPW (n = 24) HNPW (n = 24) CHM (n = 24)	Blood/VD and heavy metals (HPLC and AAS)	First trimester	Supplementation: not reported Concentrations: not clear	Cd, Cu, Zn and Co	In CHM patients, low VD concentrations and significantly elevated Cd and Fe levels were observed when compared to the HPW and HNPW groups.	Medium
[45]	Catalase activity, serum trace and heavy metal concentrations, vitamin A, D and E levels in preeclampsia (2008)	Observational	Turkey	HPW (n = 48) HNPW (n = 50) PE (n = 47)	Blood/VD and heavy metals (HPLC and AAS)	Third trimester	Supplementation: not reported Concentrations: not clear	Cd, Cu, Zn and Co	Serum levels of VD, A and E were significantly lower in the PE group compared to the HPW and HNPW groups. Likewise, the concentrations of Cd, Cu, and Fe were significantly higher in the PE group than in the HPW and HNPW groups.	Medium

AAS: Atomic Absorption Spectroscopy; Ag: silver; Al: aluminum; As: arsenic; As^3+^: arsenite; As^5+^: arsenate; AsB: arsenobetaine; Ba: barium; Be: beryllium; Cd: cadmium; Co: cobalt; Cr: chromium; Cs: cesium; Cu: copper; CHM: complete hydatidiform mole; Cr: chromium; Cu: copper; DMA: dimethylarsinic acid; ELISA: Enzyme-Linked Immunosorbent Assay; FAES: Flame Atomic Emission Spectrophotometer; Fe: iron; Hg: mercury; HNPW: healthy non-pregnant women; HPW: healthy pregnant women; HPLC: High-Performance Liquid Chromatography; ICP-MS: Inductively Coupled Plasma Mass Spectrometry; LC-HG: Liquid Chromatography Coupled with Hydride Generation; LC-MS/MS; Liquid Chromatography–Tandem Mass Spectrometry Method; Li: lithium; LIAISON: chemiluminescence immunoassay; MMA: monomethylarsonic acid; Mn: manganese; Mg: magnesium; Mo: molybdenum; Ni: nickel; Pb: lead; PE: preeclamptic women; PPTB: Premature Rupture of Membranes; PPROM: preterm premature rupture of membranes; PCR: Polymerase Chain Reaction; PTB: Preterm Birth; Rb: rubidium; Se: selenium; SG: specific gravity; Sn: tin; Sr: strontium; Th: thorium; Ti: titanium; Tl: thallium; U: uranium; UCB: umbilical cord blood; V: vanadium; VD_3_: cholecalciferol; VDD: vitamin D deficiency; VDR: polymorphisms of the vitamin D receptor gene; W: tungsten; Zn: zinc.
